# *TICRR* serves as a prognostic biomarker in lung adenocarcinoma with implications in RNA epigenetic modification, DDR pathway, and RNA metabolism

**DOI:** 10.3389/fonc.2023.1274439

**Published:** 2023-12-13

**Authors:** Xunbo Zheng, Li Han, Jun Guan, Chenteng Chen, Yue Zhang, Jiali Zhang, Yiran Zhang, Siyao Liu, Junyan Su, Mengyuan Liu, Hanxing Huang

**Affiliations:** ^1^ The School of Clinical Medicine, Fujian Medical University, Fuzhou, China; ^2^ Department of Respiratory and Critical Illness Medicine, the First Hospital of Putian City, Putian, China; ^3^ Beijing ChosenMed Clinical Laboratory Co. Ltd., Beijing, China; ^4^ Department of Cardiothoracic Surgery, the First Hospital of Putian City, Putian, China; ^5^ Department of Pathology, the First Hospital of Putian City, Putian, China

**Keywords:** lung adenocarcinoma, TICRR, cell cycle, immune feature, RNA metabolism, nomogram

## Abstract

**Purpose:**

*TOPBP1* interacting checkpoint and replication regulator (*TICRR*), a hub gene of the Cdk2-mediated initiation step of DNA replication, has been shown an essential role in tumorigenesis by accelerating the DNA replication of tumor cells.

**Methods:**

RT-qPCR was used to detect the mRNA expression of *TICRR* in LUAD tumors and adjacent normal tissues. The Cancer Genome Atlas (TCGA) and Gene Expression Omnibus (GEO) database of LUAD were acquired to analyze the critical role of *TICRR* expression in survival prognosis and clinicopathology characters in LUAD. Gene ontology (GO), Kyoto Encyclopedia of Genes and Genomes (KEGG), and gene set enrichment analysis (GSEA) were performed using the R package. The correlation of *TICRR* expression with immune cell infiltration, RNA epigenetic modification, DNA damage repair (DDR) pathway, and cell metabolism of LUAD was further explored to verify significant conclusions.

**Results:**

*TICRR* was significantly upregulated in most cancer types, including LUAD, lung squamous cell carcinoma (LUSC), and others. Cox regression analysis indicated the overexpression of *TICRR* was associated with poor survival in several cancers. In LUAD, *TICRR* expression was positively correlated with tumor stage and was increased in smoking, male, and high tumor mutational burden (TMB) patients. Enrichment analysis revealed that *TICRR* could influence tumor proliferation and prognosis via activating pathways involving cell cycle, DNA repair, DNA replication, cysteine metabolism, oxidative phosphorylation, and ubiquitin-mediated proteolysis pathways. Interestingly, high *TICRR* expression correlated with DDR pathway signature (34 genes), 37 m6A/m5C regulated genes, and some metabolism-regulated genes. Silencing the *TICRR* gene affects cysteine metabolism and modifies cancer-related pathways, with decreased cell cycle and increased B/T cell receptor signaling. Our *TICRR* risk model accurately predicts LUAD patient prognosis, validated across GEO datasets, and is integrated with clinical characteristics via a nomogram, facilitating personalized treatment strategies and enhancing patient management.

**Conclusions:**

Taken together, *TICRR* has emerged as a promising prognostic biomarker in lung adenocarcinoma (LUAD), with implications in immune activation, cell cycle regulation, RNA modification, and tumor energy metabolism. These findings suggest that *TICRR* could serve as a viable therapeutic target and a reliable prognostic indicator for LUAD.

## 1 Introduction

Global cancer data shows lung cancer is the leading cause of cancer mortality (1). Non-small cell lung cancer (NSCLC) accounts for approximately 85% of lung cancer cases based on histological classification, and the remaining 15% are small cell lung cancer (SCLC). The two most prevalent subtypes of NSCLC are lung adenocarcinoma (LUAD) and lung squamous cell carcinoma (LUSC) (2). Targeted therapy and immunotherapy have revolutionized the landscape of lung cancer treatment. Still, the 5-year survival is dropped from 92% of IA1 to 13% of IIIC due to the limited benefit population, therapy resistance, and micrometastasis after surgery (3). It is urgent to explore new prognostic biomarkers and drug targets to improve the efficacy of lung cancer treatment.

*TOPBP1* interacting checkpoint and replication regulator (*TICRR*, also known as *C15orf42*, *FLJ41618*, *MGC45866*, *SLD3*, Treslin) was first identified in Xenopus egg extracts, which collaborated with *TopBP1* in the Cdk2-mediated initiation step of DNA replication to support S phase regulation (4). The previous study has demonstrated that knockdown *TICRR* not only inhibits the initiation of DNA replication but also hinders fork progression. In p53-wild tumor cells, silencing *TICRR* suppresses DNA synthesis, leading to the accumulation of DNA damage. Consequently, this activates the ATM/CHK2-dependent p53 signaling pathway, ultimately inducing cell cycle arrest and apoptosis. Further, the *TICRR* was overexpressed in several cancers (5). Xia, S. et al. found that aberrant expression of *TICRR* could contribute to papillary renal cell carcinoma (PRCC) tumorigenesis by regulating the cell cycle (6). However, it remains unclear about the biological features and co-expressed genes of *TICRR*, which may affect the prognosis of LUAD.

Recently, RNA modifications represented by m6A and m5C have significantly impacted cancer development, progression, and prognosis (7). Alterations in the DNA damage repair (DDR) pathway, particularly in homologous recombination (HR) and mismatch repair (MMR), have frequently been identified to be therapeutic targets and prognosis biomarkers (8). In addition, as one of the essential energy supplies, tumor metabolic genes could affect tumorigenesis by regulating the metabolic pathway (9). However, no studies focus on the correlation between the above biomarkers and *TICRR*.

In this study, The Cancer Genome Atlas (TCGA)-LUAD dataset and Gene Expression Omnibus (GEO) were acquired to investigate the differential expression of *TICRR* in various cancers between tumor and normal tissues. To verify the significant association of *TICRR* expression with LUAD tumorigenesis and progression, we comprehensively analyzed its prognostic value, functional roles, and immunological characteristics. In addition, the correlation of the DNA damage repair (DDR) pathway, m6A and m5C-regulated genes, and cell metabolism-related genes with *TICRR* expression were analyzed to explore the biological mechanism of LUAD.

## 2 Methods

### 2.1 Data source

The normalized pan-cancer and normal tissue RNA expression data were obtained from the University of California, Santa Cruz (UCSC) datasets (https://xenabrowser.net/) containing 33 cancer types. The mutation data of LUAD were downloaded from TCGA (https://portal.gdc.cancer.gov/). The multiple RNA expression profiles were obtained from GEO [https://www.ncbi.nlm.nih.gov/geo/). GEO datasets (GSE72094 (10), GSE50081 (11), GSE13213 (12), GSE30219 (13), GSE41271 (14), GSE42127 (15), GSE126044 (16), and GSE210129 (17)] were enrolled to validate the prognosis value. After excluding samples without survival information, these datasets retained 398, 127, 116, 85, 182, and 133 samples, respectively. Besides serving as validation sets for *TICRR* expression and prognosis in GSE50081 and GSE30219, the remaining four datasets were also employed for external model validation. RNA-seq data from GSE126044 were used to analyze the predictive ability of the immunotherapy outcomes. The correlation of clinical characteristics and *TICRR* expression for the above TCGA and GEO datasets were shown in **Supplementary Tables 1**-**7**. The GSE210129 dataset contained the RNA-seq data of three control and six siTRESLIN samples of Hela cell line, which was used to explore the underlying biological mechanism and function.

In addition, single-cell RNA sequencing data of six LUAD patients from Bischoff, P. et al. (2021) were included to reveal the gene expression features in different cell types (18).

The m6A/m5C regulated genes were acquired from Li, D. et al. (2022) (19), 34 DDR-related genes were acquired from Carlo, M.I. et al. (20), and the metabolism gene list was obtained from Possemato, R. et al. (2011) (9).

### 2.2 Differential expression and prognosis risk analysis of *TICRR* in pan-cancer

The *TICRR* expression profile in pan-cancer was analyzed using the “limma” package. Data were excluded according to the two criteria: (i) samples with no *TICRR* expression; (ii) cancer types with less than three samples.

The clinical data of LUAD data sets were acquired in the TCGA database. The correlation analysis between *TICRR* expression level and clinicopathological characteristics of LUAD patients, such as clinical stage, gender, and smoking status, was implemented. Furthermore, Univariate analysis can be used for the initial exploration of the relationship between *TICRR* and clinical factors with prognosis, while multivariate analysis can further eliminate the influence of other confounding factors.

### 2.3 Reverse transcription-quantitative polymerase chain reaction

In addition, we validated the differential expression of *TICRR* in LUAD tissues and paired paracancerous tissue by RT-qPCR experiment. Total RNA of 16 paired frozen fresh tumor tissues and paired paracancerous tissues of LUAD was extracted using the TRIzol reagent (Invitrogen) following the manufacturer’s protocol. Reverse RNA transcription to cDNA was obtained using PrimeScript™ RT Master Mix (Takara, Shiga, Japan) according to the manufacturer’s instructions. The qPCR was performed with QuantStudio 5 Detection System (ABI, Thermo Fisher) in a 20 μl reaction mixture containing SYBR GreenII. The expression of *TICRR* was normalized to GAPDH and was analyzed using the 2^−ΔΔCT^ method. [ΔCT = CT(target gene) − CT(reference gene), ΔΔCT = ΔCT(tumor sample) − ΔCT(normal sample)]. The primer sequences are presented in **Table 1**.

**Table 1:** RT-qPCR primer sequences used in this study.

Primers	Sequences	
TICRR	Forward	CACGGGAGACGAAGAGGT
	Reverse	CTGGAACAGCAGCGGAGA
GAPDH	Forward	TGCACCACCAACTGCTTAGC
	Reverse	GGCATGGACTGTGGTCATGAG

### 2.4 Genomic alterations analysis

The mutation data of TCGA-LUAD was downloaded from the cBioPortal online tool (https://www.cbiobortal.org) (21). The “‘Maftools” package (22) was then used to visualize and analyze data of somatic mutations.

### 2.5 Immune features analysis

The Cibersort algorithm was used to quantify the infiltrate levels of 22 immune cells for each LUAD cancer sample with TCGA expression data (23). The “estimate” R package was used to calculate the immune and ESTIMATE scores of LUAD patients. A total of 33 cancers have been previously examined in the TCGA project. The tumors have been categorized into six immune subtypes as follows: C1 (wound healing), C2 (IFN-γ dominant), C3 (inflammation), C4 (lymphocyte depletion), C5 (immunologically silent), and C6 (TGF-beta dominance) (24). *TICRR* expression differences between subtypes were analyzed. In addition, the expression of immune checkpoints was applied to evaluate the relationship between *TICRR* high and low expression groups.

### 2.6 Enrichment analysis of *TICRR* and its co-expression genes in TCGA

The co-expression genes correlated to *TICRR* expression in LUAD were analyzed by Pearson’s correlation coefficient calculated by the R software. The “pheatmap” package drew the top 50 co-expression genes. Gene Set Enrichment Analysis (GSEA) and Gene Set Variation Analysis (GSVA) were conducted to investigate the biological functions of *TICRR* in tumors. The gene set’ c2.cp.kegg.v7.4.symbols.gmt’ was downloaded from MSigDB v7.5 for GSEA and GSVA (25). Gene ontology (GO) and Kyoto Encyclopedia of Genes and Genomes (KEGG) pathway enrichment analysis of *TICRR* co-expression genes were conducted using the “clusterProfiler” package (26).

### 2.7 Gene correlation and interaction analysis of *TICRR*

The Pearson correlation analysis between 34 DNA damage repair (DDR) genes, 7 Cysteine-related genes, 12 Glycolysis, and *TICRR* expression in LUAD were implemented using R software. A lollipop plot was used to show the correlation coefficient.

The online website tool GeneMANIA database (http://www.genemania.org) was utilized to find functionally similar genes for *TICRR* based on the interactions datasets from GEO and other organism-specific functional genomics datasets (27).

### 2.8 Drug sensitivity analysis of *TICRR*

Drug sensitivity information was downloaded from The Genomics of Drug Sensitivity in Cancer (GDSC) database (https://www.cancerrxgene.org/) (28). The half-maximal inhibitory concentration (IC50) of represented drug response was estimated using an R package “oncoPredict” (29). Finally, we used PubChem (https://pubchem.ncbi.nlm.nih.gov/) website to visualize the 3D structure of sensitive drugs.

### 2.9 Target miRNA prediction and competing endogenous RNA network construction

The screening criteria were a mammal, human, hg19, strict stringency (≥5) of CLIP-Data, and with or without data of Degradome-Data. The target miRNA was predicted using two online databases, miRDB (http://mirdb.org/miRDB/) and miRWalk (http://mirwalk.umm.uni-heidelberg.de/). Target miRNAs of *TICRR* were defined as miRNAs found in both databases, and the target score was ≥0.8. The ceRNA network of miRNA–lncRNA–circRNA interaction was constructed by StarBase v2.0 (https://starbase.sysu.edu.cn/index.phpStarBase) (30). The Cytoscape was applied to visualize the ceRNA networks.

### 2.10 Single-cell RNA sequencing analysis

Six single-cell RNA sequencing data of LUAD from the Bischoff cohort were enrolled to analyze the expression features of *TICRR* in the TME. The uniform manifold approximation and projection (UMAP) method (31) was used for cluster visualization, and the “SingleR” package was used for cluster annotation. “FeaturePlot” and “VlnPlot” were used to visualize gene expression.

### 2.11 Statistical analysis

Univariate and multivariate Cox regression analyses were conducted to evaluate the risk factors and prognostic value of *TICRR* in cancers. Statistical analysis utilized R software (version 4.2.1) and its corresponding packages. The correlation coefficient was calculated using Pearson correlation analysis. The Wilcoxon test was used to investigate the difference between the groups. All the P-values were two-sided, and the results were considered statistically significant when the P-values were less than 0.05. Survival curves for OS were compared using the Kaplan-Meier (KM) method. The prognosis performance was evaluated by receiver operating characteristic (ROC) curves and the area under the ROC curve (AUC) value.

## 3 Results

### 3.1 Expression and prognostic significance of *TICRR* in LUAD and pan-cancer

**Figure 1:**
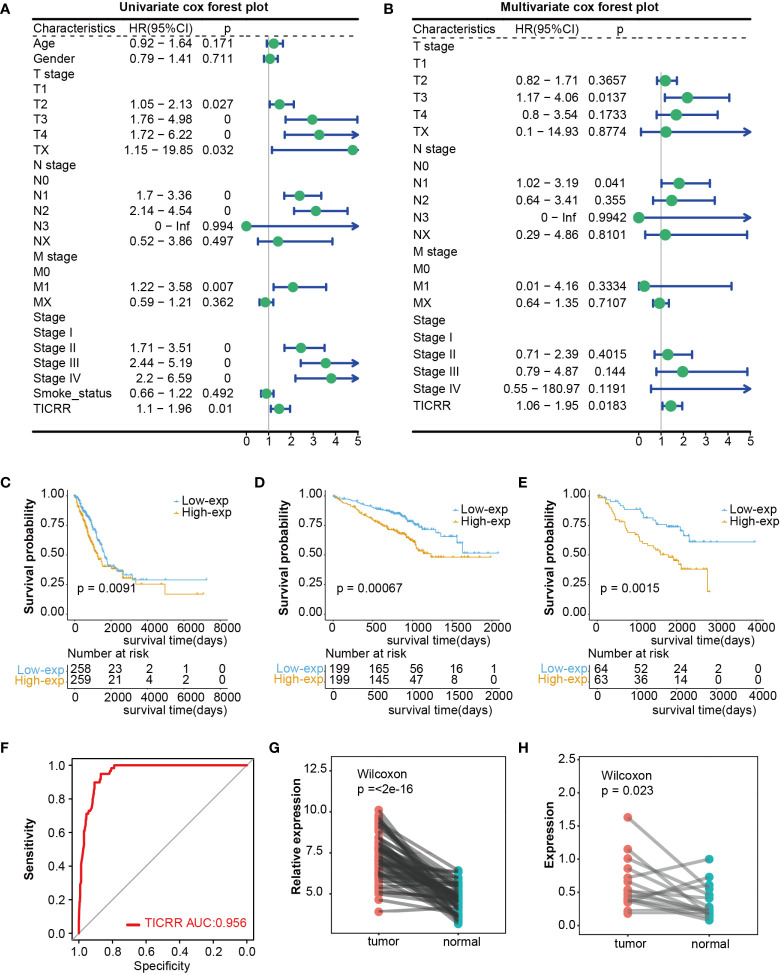
Differential expression and prognosis value of *TICRR* in cancers. **(A)** The univariate Cox regression analysis for LUAD in TCGA dataset. This analysis serves as an initial exploration of the relationship between *TICRR*, clinical factors, and prognosis. **(B)** The multivariate Cox regression analysis for LUAD in TCGA dataset, which further eliminates the influence of other confounding factors. **(C–E)** Survival analysis for LUAD in TCGA, GSE72094, and GSE50081 datasets based on the *TICRR* expression. Green represents the low-exp group. Blue represents the high-exp group. **(F)** Diagnostic ROC analysis with the AUC of *TICRR* in LUAD. **(G, H)** Differences of *TICRR* expression between LUAD tumor and normal tissues in TCGA and 16 clinical LUAD samples.

Firstly, comparing TCGA tumors and normal tissues indicated considerably different *TICRR* expression across most cancer types, including LUAD, LUSC, and others. When compared to normal samples, a notable increase in *TICRR* expression was observed in 17 different cancer types, The expression level of *TICRR* is significantly upregulated in LUAD (p<0.0001)(**Supplementary Figure 1**). These findings strongly indicate that *TICRR* expression is consistently upregulated in various cancer types, highlighting its potential pivotal role in cancer diagnosis. Cox regression analysis revealed that overexpressed *TICRR* could indicate poor prognosis in most cancer types (**Supplementary Figure 2**), and univariate Cox regression indicated *TICRR* was a risk factor for the overall survival in LUAD [HR=1.47, 95% CI (1.1-1.96), *p*=0.01](**Figure 1A**). Multivariate Cox regression analysis further confirmed the prognosis significance of *TICRR* in LUAD [HR=1.44, 95% CI (1.06-1.95), *p*=0. 018] (**Figure 1B**). The detailed univariate and multivariate Cox regression analysis results were displayed in **Supplementary Table 8**. Furthermore, survival analysis across pan-cancer was shown in **Supplementary Figure 3**, and TCGA-LUAD patients could be classified into high and low-exp groups according to the median expression of *TICRR* (**Figure 1C**). Two more LUAD GEO cohorts were used to conduct the survival analysis to confirm the prognostic effect of *TICCR* expression. Patients in both GSE72094 (*p*=0.00067) and GSE50081 (*p*=0.0015) could be divided into differential groups based on the median expression of *TICRR* (**Figures 1D, E**). *TICRR* expression could also indicate prognosis in another 4 GEO datasets (GSE13213, GSE30219, GSE41271, and GSE42127) (**Supplementary Figures 4A-D**). Receiver operating characteristic (ROC) curve was used to determine the efficacy of gene expression data in predicting disease groups. Different AUC cutoffs were considered to indicate high diagnostic accuracy (AUC: 1.0-0.9), relative diagnostic accuracy (AUC: 0.9-0.7), or low diagnostic accuracy (AUC: 0.7-0.5), we found that *TICRR* could accurately differentiate the LUAD from the normal, with an AUC of 0.956 (**Figure 1F**). RT-qPCR validation revealed that *TICRR* mRNA expression was significantly higher in 16 pairs of LUAD tissues than in paracancerous tissues (*p*=0.023) (**Figure 1H**), and the experimental data were presented in **Supplementary Table 9**. The validation results were consistent with the expression difference of *TICRR* in TCGA datasets (*p*<0.001)(**Figure 1G**).

### 3.2 Associated of *TICRR* with mutational landscape and clinical feature in TCGA-LUAD

**Figure 2:**
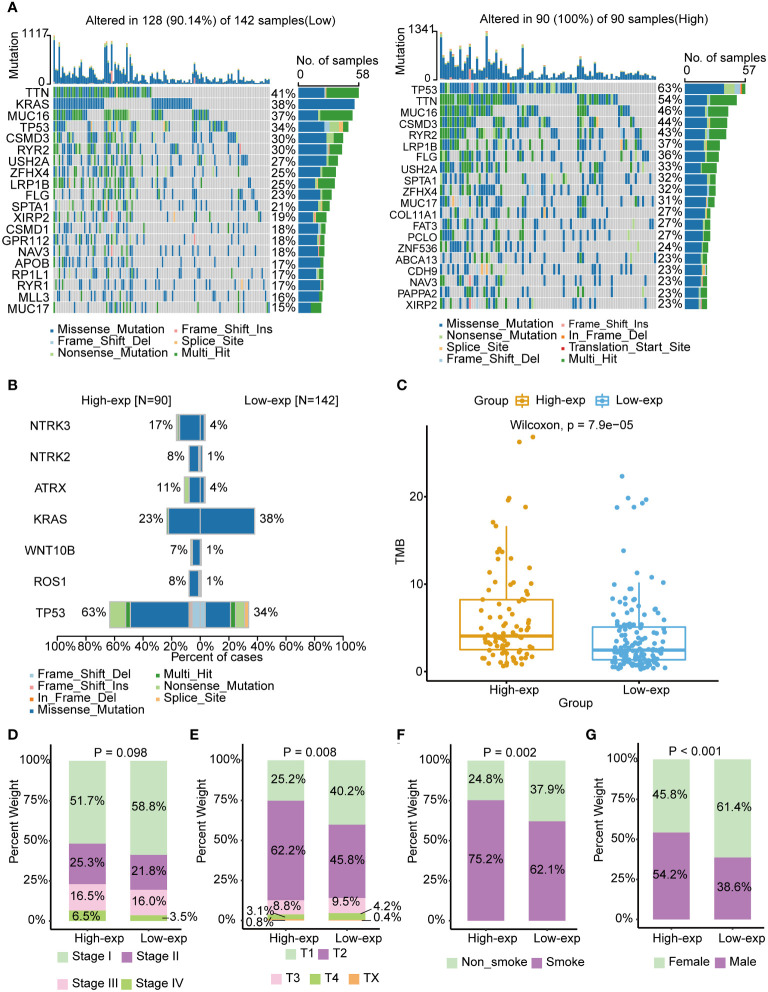
Associated of *TICRR* expression with the mutational landscape, clinical characters, and TMB in LUAD. **(A)** The mutational landscape of *TICRR* low (left) and high(right) expression group in LUAD. The top bar chart illustrates the count of mutations per sample. The rightmost bar chart represents the variety of mutations in each gene. **(B)** Significantly different mutant genes between *TICRR* high and low expression groups. **(C)** Correlation of tumor mutational burden (TMB) with *TICRR* expression in LUAD. **(D–G)** Correlation of tumor stage, T stage, smoking, and gender with *TICRR* expression in LUAD.

Previous studies have identified that oncogene and suppressed gene mutations and clinical characteristics had been recognized as risk factors in LUAD, such as *EGFR*/*KRAS* gene alterations and smoking (2). We subsequently investigated the association of *TICRR* with these established prognosis factors. The mutation landscape showed noticeably distinct alterations across a differential expression of the *TICRR*. Missense mutation mainly caused by SNV was the major type of mutations. The most frequently mutated genes were roughly the same in high and low exp tumors. *TP53*/*TTN*/*MUC16* were more frequently mutated in the *TICRR* overexpressed group, while *KRAS* was more commonly mutated in the *TICRR* downregulated group (**Figure 2A**). Fisher’s test revealed significant differences in gene alterations between the high-expression and low-expression groups, including NTRK3, NTRK2, ATRX, KRAS, WNT10B, ROS1, and TP53(P<0.05, **Figure 2B**). The *TICRR* overexpressed group had increased TMB levels (P<0.001, **Figure 2C**). In addition, *TICRR* was determined to closely relate to the Tumor stage, T stage, smoking status, and gender of LUAD patients (**Figures 2D–G**). Although the correlation between the *TICRR* expression and staging did not reach statistical significance, it’s noteworthy that the low-exp group had a higher proportion of stage I compared to the high-exp group. In contrast, the high-exp group had lower proportions of stage II, III and IV than the low-exp group. Meantime, a significant difference was observed in terms of T-stage between the high-exp and low-exp groups (p=0.008). The *TICRR* overexpressed group had more smokers (*p*=0.002) and male patients (*p*<0.001).

### 3.3 Co-expression genes enrichment analysis

**Figure 3:**
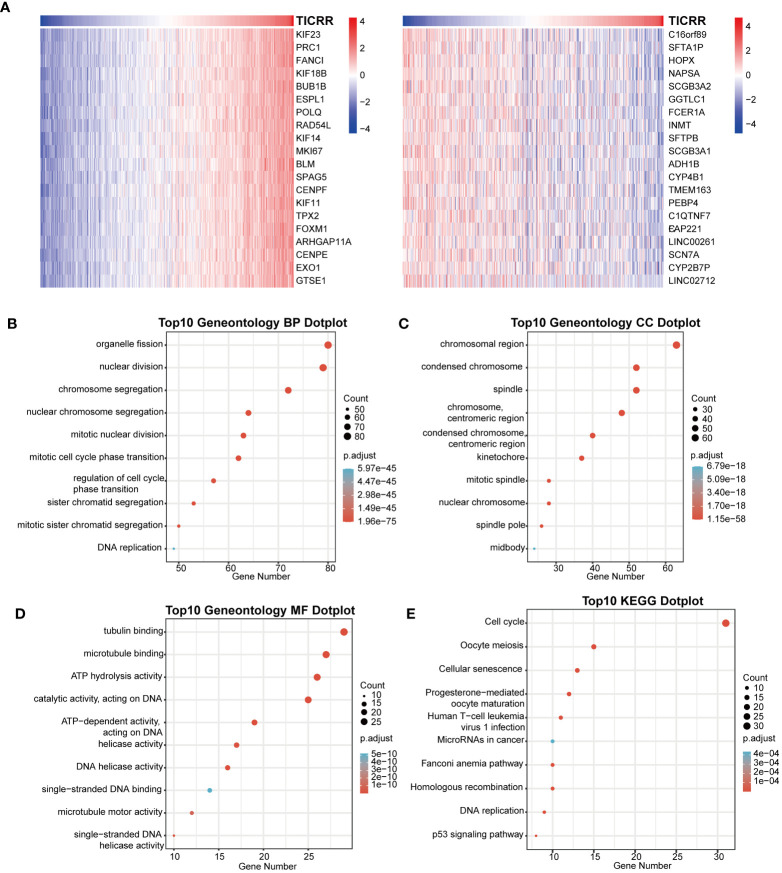
Correlation of co-expression genes with the expression of *TICRR*. **(A)** The heatmap showed the top 50 co-expression genes significantly positively (left) and negatively (right) correlated with *TICRR* expression. **(B–E)** Enrichment analysis of gene ontology (GO) terms and Kyoto Encyclopedia of Genes and Genomes (KEGG) pathways for top 200 *TICRR* co-expression genes.

**Figure 4:**
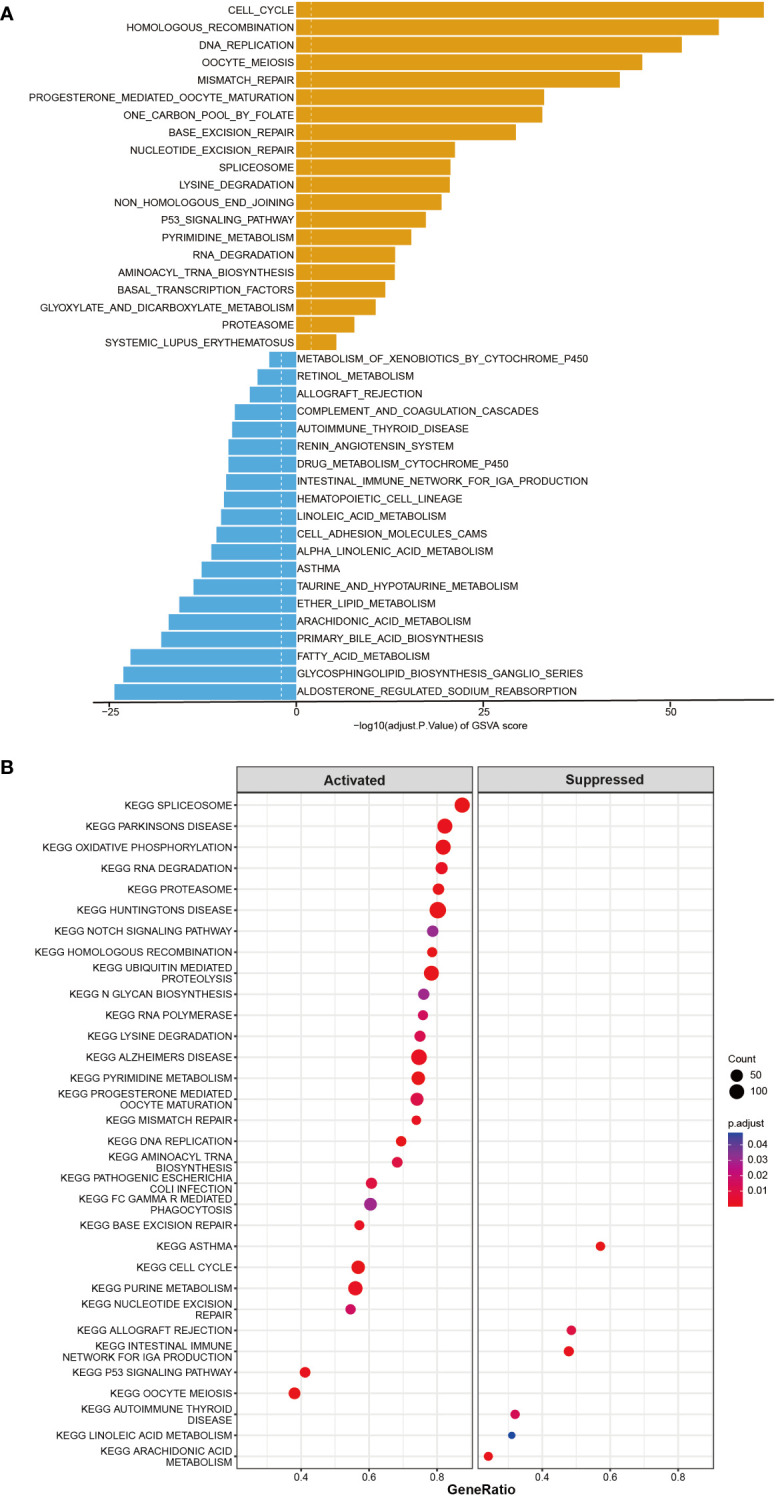
GSVA and Gene Set Enrichment Analysis (GSEA) of the *TICRR* in LUAD. **(A)** GSVA analyzed the top20 biological pathways of *TICRR* high and low expression groups. Orange signifies a notable enrichment of this biological pathway in the high-expression group, whereas blue indicates a significant enrichment in the low-expression group. **(B)** GSEA showed the most enriched gene sets of all detected genes in *TICRR* high and low expression groups.

Furthermore, the co-expression genes with *TICRR* were confirmed using the TCGA-LUAD dataset. There were 22460 genes positively correlated with the RNA expression of *TICRR*, and 1807 genes were negatively correlated with the RNA expression of *TICRR* (P<0.05). The top 50 positively (**Figure 3A**, left) and negatively (**Figure 3A**, right) associated genes with *TICRR* were displayed, and the detailed correlation statistics between *TICRR* and other genes were listed in **Supplementary Table 10**. Next, the GO annotation and KEGG pathway enrichment using the top 200 co-expression were performed to explore the biological function of *TICRR* and co-expression genes. The detailed enrichment analysis results were listed in **Supplementary Tables 11**, **12**. The results of GO-BP, GO-CC, GO-MF, and KEGG pathways only showed the top 10 terms (**Figures 3B–E**). These genes were involved in the cell cycle, homologous recombination pathway, organelle fission, tubulin binding, and chromosomal region-relative activities. Then GSVA and Gene Set Enrichment Analysis (GSEA) of the *TICRR* expression was performed, and the GSVA results were presented in **Supplementary Table 13**. The bar plot showed that upregulated *TICRR* expression was positively related to the cell cycle, homologous recombination pathway, DNA replication, and some energy metabolism pathway (**Figure 4A**), consistent with the co-expression genes enrichment analysis. *TICRR* gene could also activate ubiquitin-mediated proteolysis and NOTCH signaling pathways (**Figure 4B**).

### 3.4 The correlation between tumor immune microenvironment, immunotherapy response, and *TICRR* expression

**Figure 5:**
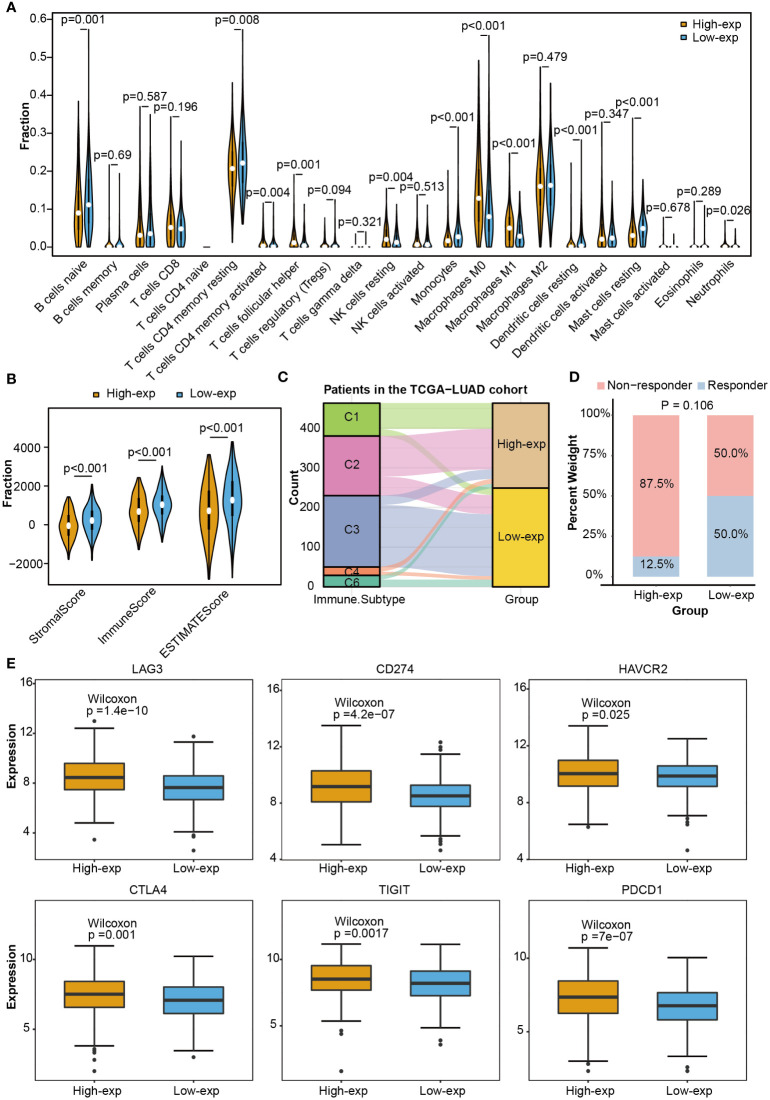
Immune correlation analysis between high and low expression groups according to *TICRR* expression in TCGA-LUAD. **(A)** Comparing 22 immune cell fractions between high and low expression groups. **(B)** Immune score, **(C)** immune subtype, **(D)** immunotherapy response, and **(E)** differential expression of immune checkpoint genes between high and low expression groups.

As the above proved, TCGA-LUAD patients could be stratified into high-exp and low-exp groups according to *TICRR* median expression. To clarify the impact of the *TICRR* gene on the tumor immune microenvironment (TIME), immune score, immune subtype, immune infiltration, and single-cell public dataset enrichment analysis were conducted using the TCGA-LUAD dataset. Estimate analysis showed *TICRR* high expression group was characterized by a significantly low immune score and stromal score (*p*<0.001) (**Figure 5B**). The fraction of 22 immune cells calculated using the CIBERSORT algorithm revealed the infiltration of T cells follicular helper, NK cells resting, Macrophages M0, and Macrophages M1 were higher in the high-exp group (*p*<0.01). While the infiltration level of T cells CD4 memory resting, Monocytes, Dendritic cells resting, and Mast cells resting were higher in the low-exp group (*p*<0.01) (**Figure 5A**). Immune subtype analysis showed the C3 subtype (inflammatory) and C6 (TGF-b dominant) were primarily enriched in the low-exp group (**Figure 5C**). Six immune checkpoint genes were expressed more highly in the high-exp group than those in the low-exp group according to the *TICRR* expression level (P<0.05) (**Figure 5E**). In the GSE126044 dataset, patients could be classified into eight high-exp and eight low-exp patients. Of these 11 immunotherapy non-responders, seven were in the high-exp group, and four were in the low-exp group (*p*=0.106) (**Figure 5D**). Subsequently, single-cell analysis was performed for six LUAD patients (32). UMAP dimensionality reduction was achieved to show the distribution of the different cell types: T cells, macrophage, epithelial cells, B cells, iPS cells, tissue stem cells, and endothelial cells (**Supplementary Figure 5A**). *TICRR* was mainly enriched in macrophages and T cells (**Supplementary Figure 5B**).

### 3.5 Correlations of *TICRR* expression with DDR-pathway and energy metabolism in LUAD

**Figure 6:**
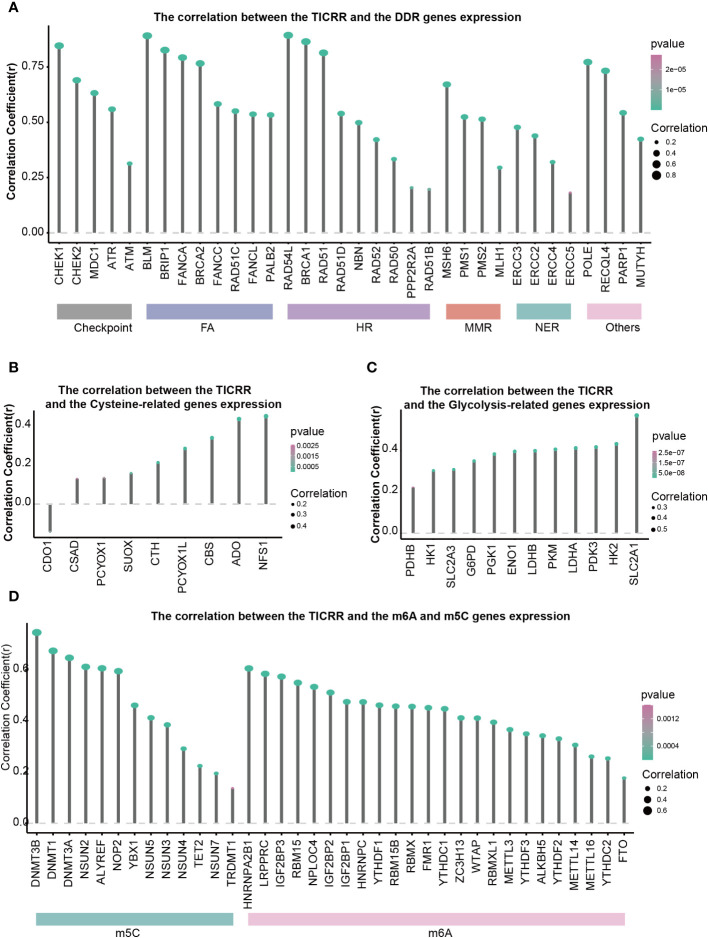
Correlations of *TICRR* expression with important biological regulation and pathways. Correlation of *TICRR* expression with **(A)** 34 DDR-related genes, **(B)** 9 Cysteine-related genes, **(C)** 12 Glycolysis-related genes, and **(D)** 37 m6A/m5C regulated genes in LUAD.

DNA damage repair (DDR) contributes to maintaining DNA integrity, cysteine is associated with antioxidant capacity, and glycolysis is involved in energy metabolism. All of these are crucial factors in the development of LUAD. The correlation between 34 DDR genes, 9 Cysteine-related genes, 12 Glycolysis, and *TICRR* expression in LUAD were shown in **Figures 6A-C**. All of the 34 DDR-related genes were positively correlated with *TICRR* expression, especially *RAD54L*, *BLM*, *BRCA1*, *CHEK1*, *BRIP1*, *RAD51*, *FANCA*, *POLE*, *BRCA2* (Correlation Coefficient>0.75) (**Figure 6A**). These significantly correlated DDR genes were mainly concentrated in the Fanconi anemia (FA), HR, and checkpoint pathways. For energy metabolism, Cysteine and Glycolysis metabolism-related genes were also positively related to *TICRR* expression (**Figures 6B, C**). Furthermore, modification of m6A/m5C plays an important role in the development of LUAD. *TICRR* expression was significantly correlated with m6A/m5C regulators, and the top 5 were *DNMT3B*, *DNMT1*, *DNMT3A*, *NSUN2*, and *ALYREF* (Correlation Coefficient>0.6) (**Figure 6D**). These results suggest that *TICRR* may be closely related to the DNA damage repair, cysteine, glycolysis, m6A/m5C modification of LUAD.

### 3.6 Drug sensitivity analysis and drug prediction

**Figure 7:**
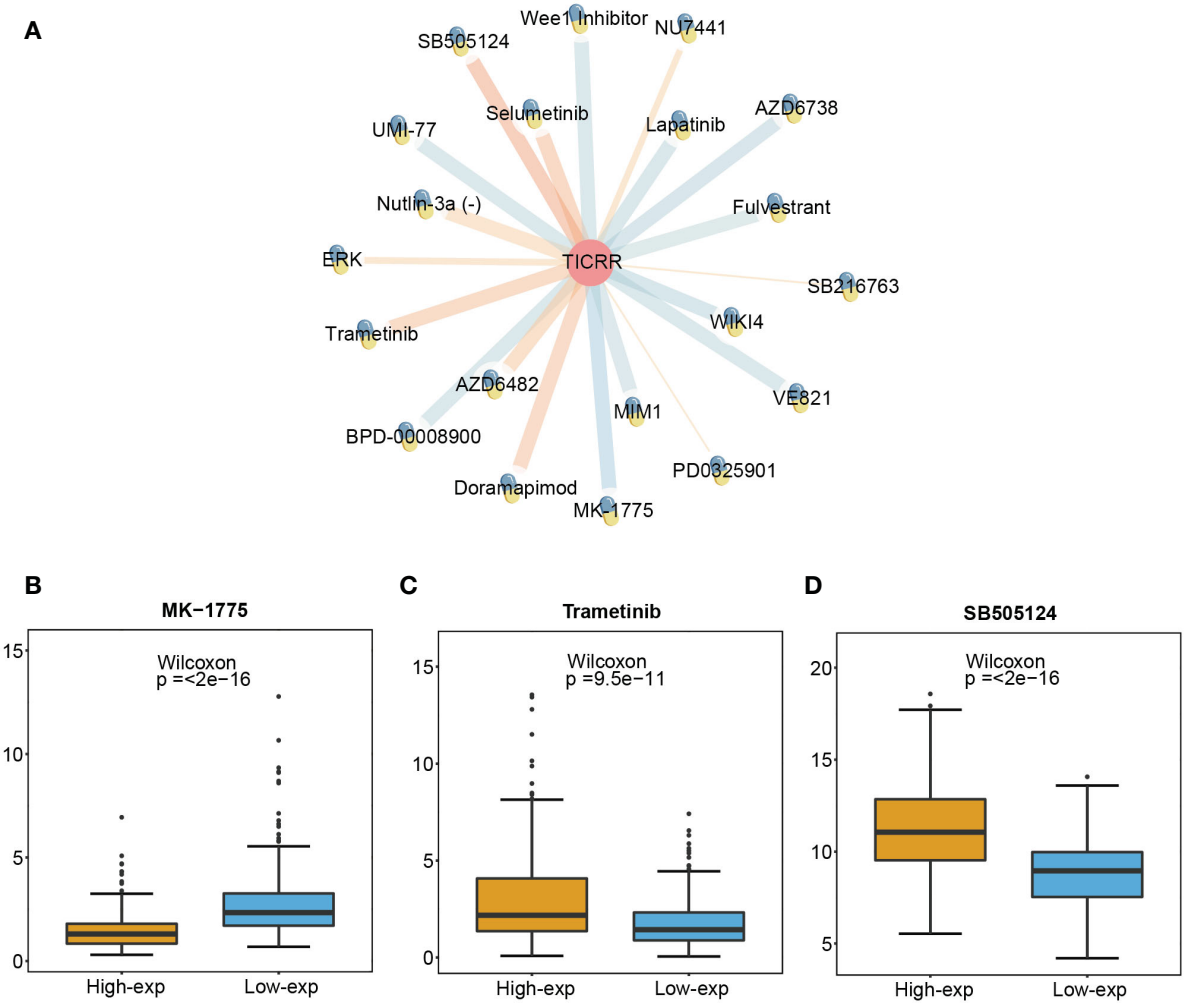
Screened drugs for LUAD treatment. **(A)** The network plot shows the correlation between GDSC drug sensitivity and *TICRR* expression. The orange lines represent a positive correlation, and the blue lines represent a negative correlation, with thicker lines indicating a more significant correlation. The different IC 50 values of **(B)** MK-1775, **(C)** Trametinib, and **(D)** SB505124 in *TICRR* high and low expression patients with LUAD.

To investigate the prediction value of *TICRR* for drug therapy, we analyzed the correlation between drug sensitivity and *TICRR* expression using the genomics of drug sensitivity in cancer (GDSC) database, and half-maximal inhibitory concentration (IC50) was used as an indicator for drug sensitivity. As we know, drug sensitivity increases as the IC50 value decreases. The top 10 significantly positively and negatively sensitive drugs correlated with *TICRR* expression were shown in **Figure 7A**, and the complement correlation results were in **Supplementary Table 14**. The *TICRR* upregulated patients exhibited decreased IC50s for MK-1775 (*p ≤* 2e−16, **Figure 7B**), a WEE1 inhibitor that targets the cell cycle pathway, suggesting that MK-1775 could help patients who are at high-exp due to *TICRR* expression. Oppositely, patients in the low-exp group were more sensitive to Trametinib (*p*= 9.5e−11, **Figure 7C**) and SB505124 (*p ≤* 2e−16, **Figure 7D**), revealing these patients might benefit from MEK and TGFβR inhibitors.

### 3.7 Interacting genes of *TICRR*

**Figure 8:**
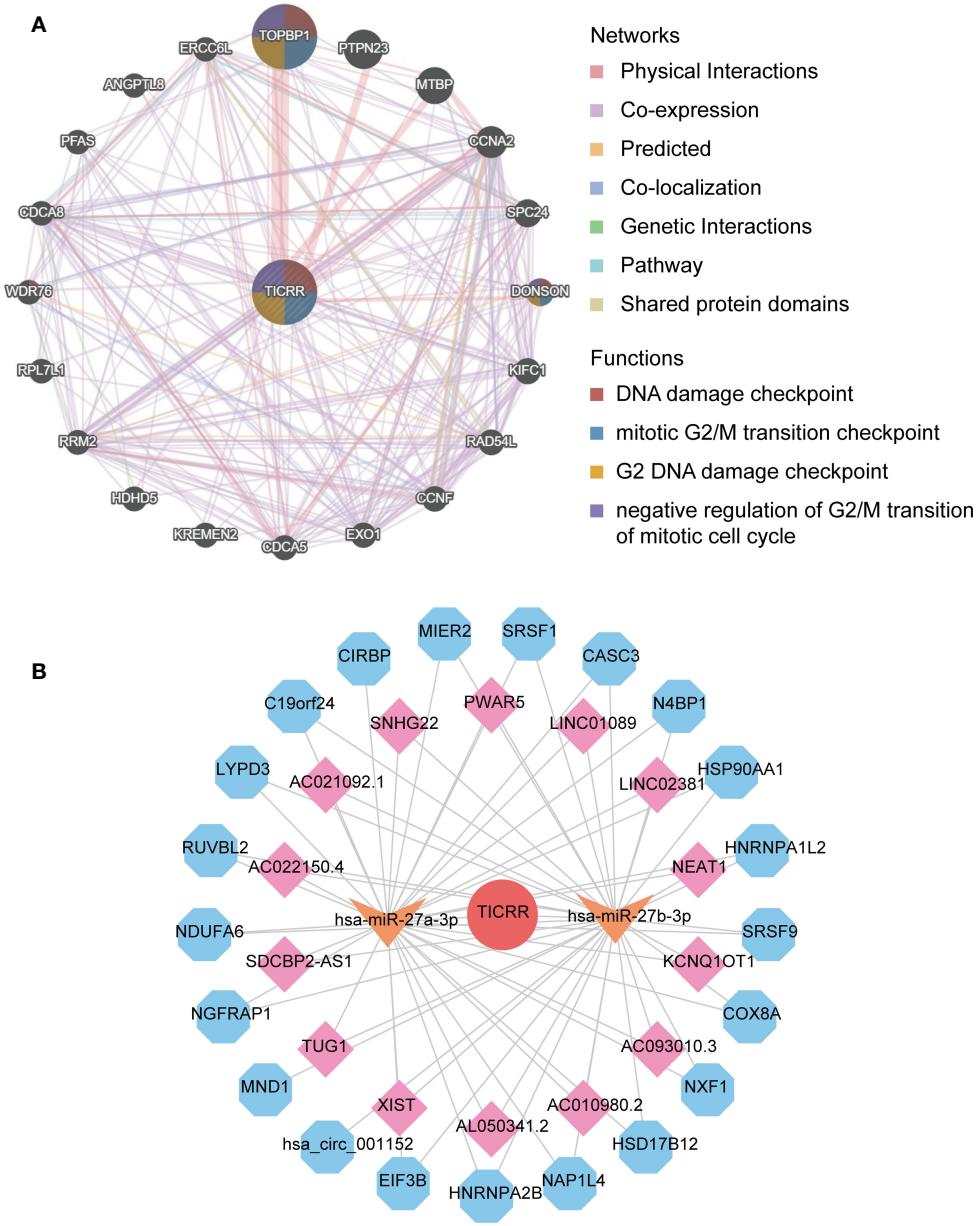
The interaction network of *TICRR* in LUAD. **(A)** gene-gene interaction network of *TICRR* from GeneMANIA. **(B)** ceRNA networks of *TICRR* (the red circle represents the hub gene *TICRR*, the orange arrow represents the miRNAs, the pink square represents the lncRNAs, and blue hexagons represent the circRNAs.

Finally, the interacting genes with *TICRR* in LUAD were identified using the GeneMANIA website tool. The top 20 associated genes and 330 links of interaction were presented in the network (**Figure 8A**). *TOPBP1* was the strongly connected gene with *TICRR*, followed by *PTPN23*, *MTBP*, *CCNA2*, and *DONSON*. The most functionally similar genes to *TICRR* were *TOPB1* and *DONSON*, which were all engaged in the DNA damage checkpoint, mitotic G2/M transition checkpoint, G2 DNA damage checkpoint, and negative regulation of G2/M transition of the mitotic cell cycle (*p*<0.05).

The ceRNA network displayed the complicated interaction within target miRNAs, lncRNAs, and circRNAs of *TICRR* (**Figure 8B**). In addition, target miRNAs of *TICRR* were predicted using multiple miRNAs database, and three miRNAs were finally screened, including hsa-miR-27a-3p, hsa-miR-9985, and hsa-miR-27b-3p (target score≥0.8). However, only two target miRNAs, hsa-miR-27a-3p and hsa-miR-27b-3p, can be retrieved in StarBase to predict their circRNAs and lncRNAs. As a result, 14 lncRNAs and 21 circRNAs correlated with the two target miRNAs of *TICRR*.

### 3.8 Function exploration when siRNA against *TICRR* in cancer cell line

**Figure 9:**
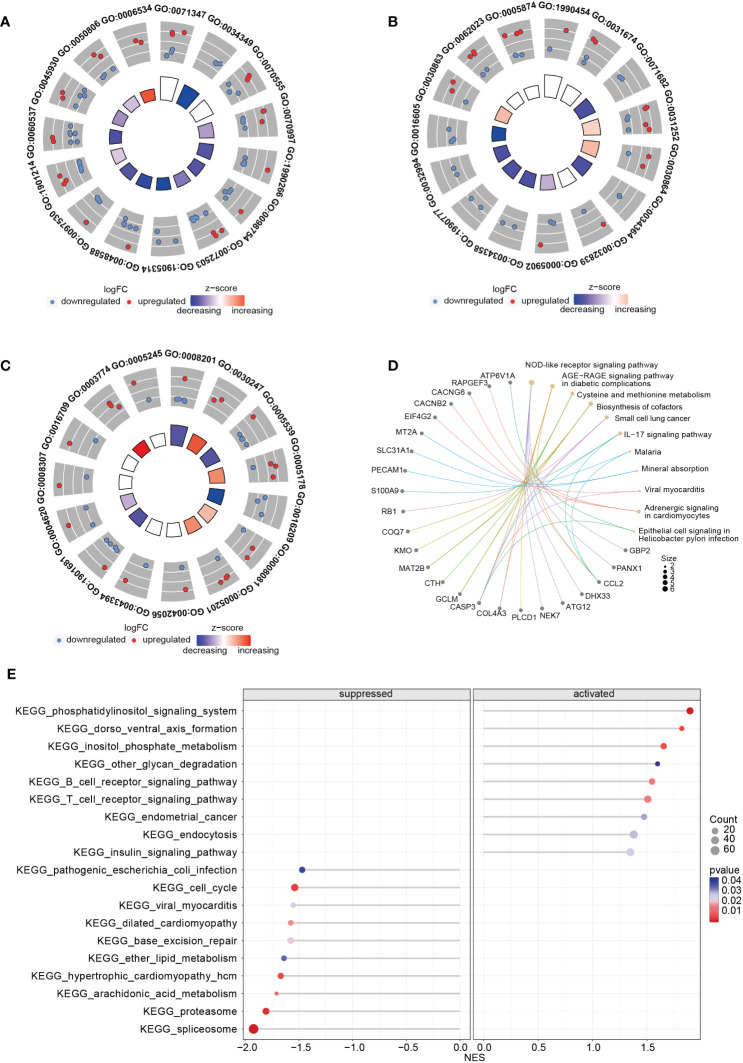
GO and KEGG enrichment analysis using the RNA expression profiles in siTRESLIN and siCTR HeLa cells. The trigram array plot demonstrated the GO enrichment analysis of BP **(A)**, CC **(B)**, and MF **(C)** based on DEGs between siTRESLIN and siCTR Hela cells. The red dots represent up-regulated genes, the blue dots represent down-regulated genes, and the inner circle is a z-score, which is not a conventional statistical z-score and only gives an indication that a term is more likely to be lower (negative) or higher (positive). **(D)** Gene-concept network shows the enriched KEGG pathway based on DEGs and the linked genes involved in these pathways. **(E)** The bubble plot exhibited KEGG pathway enrichment of KEGG pathway of siTRESLIN and siCTR HeLa cells. The colors of the lines and dots represent the P-value of the enriched KEGG pathway.

Upon comparing the RNA expression profiles of siTRESLIN and siCTR HeLa cells, we identified 126 differentially expressed genes (DEGs) between the two groups. GO enrichment analysis based on these DEGs (**Figures 9A–C**) revealed significant downregulation of processes related to PML body (GO:0016605), glial cell apoptotic process (GO:0034349), semi-lunar valve development (GO:1905314), and antioxidant activity (GO:0016209) upon *TICRR* gene silencing. Conversely, *TICRR* gene suppression led to a significant upregulation of processes related to cortical actin cytoskeleton (GO:0030864), axoneme (GO:0005930), cysteine metabolic process (GO:0006534), polysaccharide binding (GO:0030247), and integrin binding (GO:0005178) processes. KEGG enrichment analysis demonstrated several significantly enriched KEGG pathways, including NOD−like receptor signaling pathway, Cysteine and methionine metabolism, Biosynthesis of cofactors, Small cell lung cancer, IL−17 signaling pathway, Adrenergic signaling in cardiomyocytes pathways (**Figure 9D**). We also performed a GSEA analysis between siTRESLIN and siCTR HeLa cells (**Figure 9E**). When *TICRR* gene was silenced, spliceosome pathway, cell cycle pathway, arachidonic acid metabolism pathway, hypertrophic cardiomyopathy pathways were significantly decreased, while phosphatidylinositol signaling system, dorso ventral axis formation, inositol phosphate metabolism, B cell receptor signaling pathway, and T cell receptor signaling pathway. These findings revealed that the *TICRR* gene was involved in the multiple signaling transduction and metabolism processes, and played an important roles in cell cycle pathway.

### 3.9 Prognosis value of *TICRR*-related gene signature

Next, we found 86 overlap genes between 24267 *TICRR* co-expression genes and 126 siTICRR DEGs. According to the univariate and multivariate Cox regression analysis, five genes were significantly associated with the survival in LUAD, including *KIAA1549L*, *GPNMB*, *MAD2L1*, *COL4A3*, and *KRT81*. Based on the multivariate coefficient and the RNA expression value, we development a risk model to predict the survival status of LUAD. The formal of risk scores were as follows:


$$\text{Risk score}=\;\sum_{\text{i}=1}^{\text{m}}{\text{Expression}}_{\text{i}}\times {\text{Coefficient}}_{\text{i}}$$

The m is the number of signature genes for constructing the model; the “Expression_i_” indicates the expression value of signature gene “I” in the sample; the Coefficient_i_is the multivariate Cox regression coefficient of gene i.

**Figure 10:**
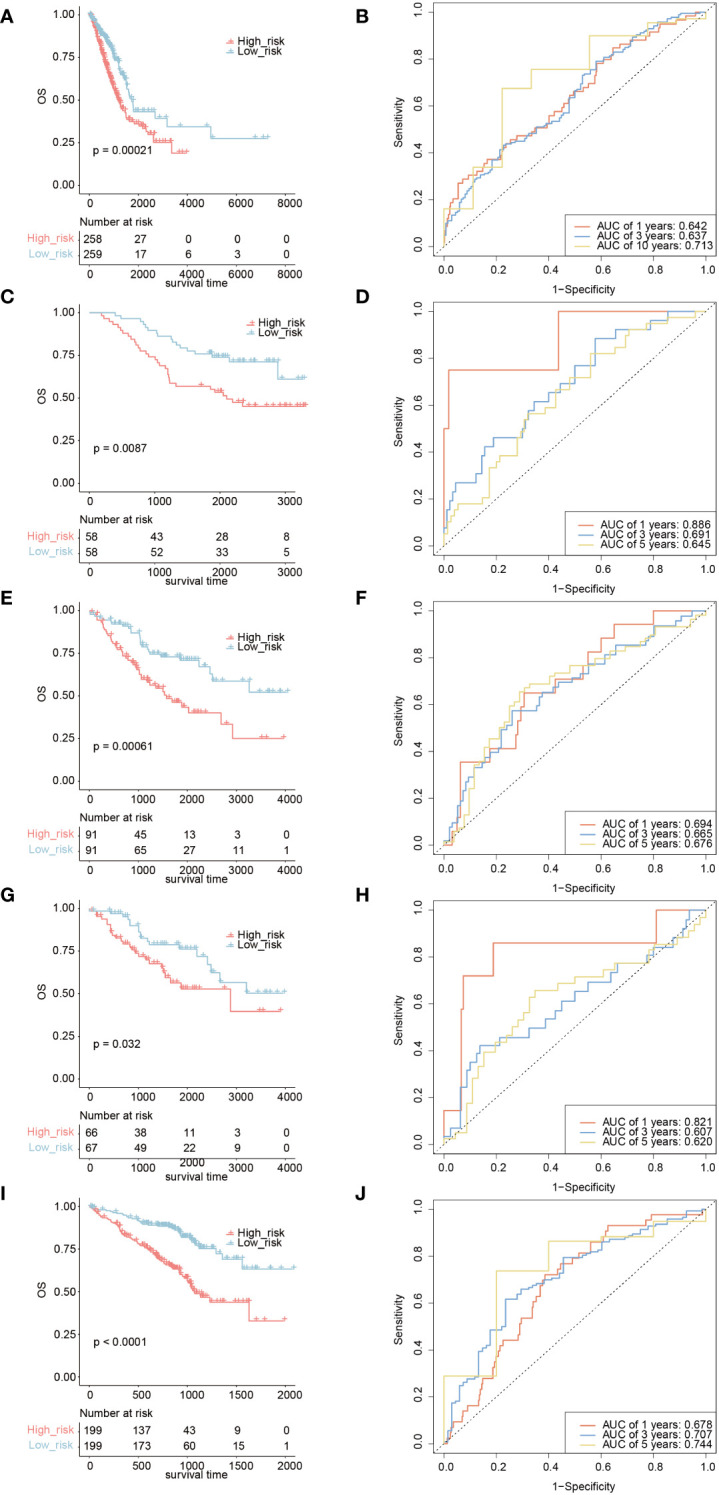
Survival analysis and AUC assessment of *TICRR* risk model in LUAD. Survival analysis for LUAD in TCGA **(A)**, GSE13213 **(C)**, GSE41271 **(E)**, GSE42127 **(G)**, and GSE72094 **(I)**. The predicting AUC assessment of *TICRR* risk model for OS survival in TCGA **(B)**, GSE13213 **(D)**, GSE41271 **(F)**, GSE42127 **(H)**, and GSE72094 **(J)**.

Based on the *TICRR* risk scores, we successfully classified TCGA-LUAD patients into high- and low-risk groups, with the high-risk group exhibiting a significantly worse prognosis compared to the low-risk group (P=0.00021, AUC=0.713) (**Figures 10A, B**). Moreover, the *TICRR* risk model effectively stratified patients in GSE13213 (P=0.0087) (**Figure 10C**), GSE41271 (P=0.00061) (**Figure 10E**), GSE42127 (P=0.032) (**Figure 10G**), and GSE72094 (P<0.0001) (**Figure 10I**), with optimal AUC values of 0.713 (**Figure 10D**), 0.694 (**Figure 10F**), 0.821 (**Figure 10H**), and 0.744 (**Figure 10J**), respectively. These results highlight the robust performance and generalizability of the *TICRR* risk model across different datasets, affirming its potential as a reliable prognostic tool in LUAD.

**Figure 11:**
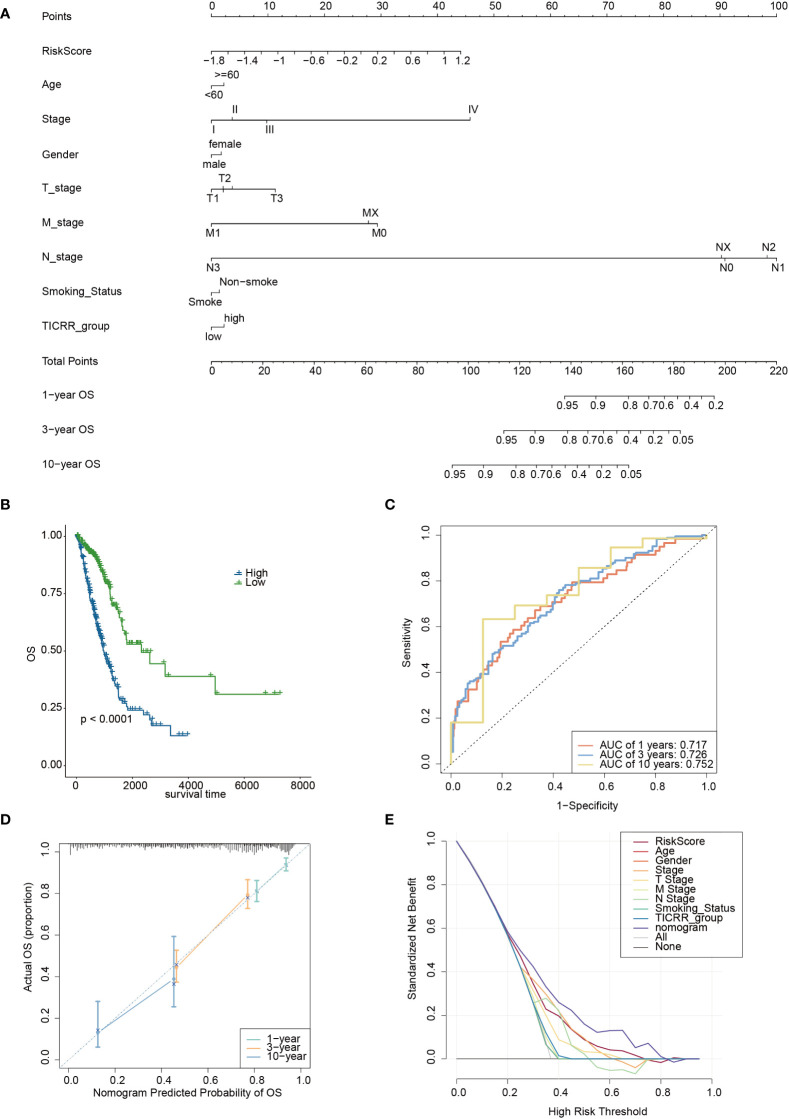
The development of a nomogram based on the *TICRR* risk model and clinical characteristics in TCGA-LUAD cohort. **(A)** A nomogram based on age, stage, gender, smoking status, *TICRR* risk groups for 1-, 3- and 101-year OS predictions. **(B)** Survival analysis of *TICRR* nomogram model in LUAD. **(C)** Predicting AUC assessment of *TICRR* nomogram model in LUAD. **(D)** Calibration curves for testing the agreement between 1-, 3- and 10-year predicted overall survival and actual observations in the EAS cohort. **(E)** DCA curve of the nomogram in LUAD.

Furthermore, we constructed a nomogram based on the *TICRR* risk scores and clinical characteristics using multivariate Cox regression analysis (**Figure 11A**). The survival analysis demonstrated significantly better OS benefits for patients in the nomogram-low group compared to the nomogram-high group (P<0.0001) (**Figure 11B**), with an optimal predicting AUC of 0.752 (**Figure 11C**). Calibration curves further revealed the nomogram’s predictive accuracy for estimating the 1-, 3-, and 10-year survival rates (**Figure 11D**). Additionally, the decision curve analysis (DCA) results confirmed the nomogram’s robustness and optimal predictive ability (**Figure 11E**).

## 4 Discussion

Cancer cells are known for their ability to sustain proliferation, and the cell cycle plays a crucial role in their development (33). The cell cycle control pathway, which includes G1-S transcription, replication stress, DNA damage, CDKs, and the mitotic checkpoint, promotes cancer cell proliferation to maintain its viability. Our research has illuminated that *TICRR* is highly expressed in tumor tissues compared to normal tissues. The study shows that *TICRR* was densely connected with m6A/m5C regulation, DDR-pathway transportation, and cancer metabolism, implying close functional interaction within them. *TICRR* demonstrates excellent prognosis value in LUAD.

Tumorigenesis involves abnormal alterations. *TICRR* expression is significantly upregulated in most cancer types and is closely related to survival prognosis. The high expression of *TICRR* has been proposed to predict poor clinical outcomes in papillary renal cell carcinoma (PRCC) (34). For most cancer types, such as LUAD, *TICRR* could predict poor prognosis accurately. The systematic analysis implied the oncogene role of *TICRR*, which could be a potential prognosis indicator in LUAD. We also find the high expression of *TICRR* was related to the activation of the cell cycle pathway, homologous recombination pathway, DNA replication pathway, some energy metabolism pathways, ubiquitin-mediated proteolysis, and NOTCH signaling pathway, which to some extent have synergistic effects in promoting LUAD tumorigenesis and progression. In summary, blocking the *TICRR* expression may improve the therapy efficiency for LUAD patients.

The *TICRR*-upregulated LUAD patients exhibit a significantly suppressed immune response status, indicated by low immune scores and high expression levels of immune checkpoint genes, leading to immune escape. Single-cell RNA-seq analysis demonstrates that *TICRR* is mainly enriched in macrophages and T cells. We speculate that overexpression of *TICRR* may reduce the anti-tumor effect by inhibiting the mature differentiation and expression of immune infiltration T cells and macrophage cells. However, the expression was lower in other cell populations. This differential expression may imply that *TICRR* has different functions and roles in different cell types. High *TICRR* expression LUAD patients show enrichment of the C1 and C2 immune subtype, associated with a high proliferation rate (35). However, immunotherapy may not be a good choice for these patients with *TICRR* upregulation.

*TICRR* is a pivotal gene in the Cdk2-mediated initiation step, crucial for DNA replication and epigenetic control (36). Furthermore, correlation analysis reveals positive association between *TICRR* expression and m6A/m5C-related genes. m5C-regulated genes are closely related to the cell cycle pathway, consistent with the KEGG enrichment in the *TICRR* overexpression group. *NSUN2*-driven RNA methylation helps to adapt cell cycle progression to early stress responses and links protein synthesis to cellular metabolism (37). Low expression of *DNMT3B* was associated with a better prognosis in LUAD (38). Thus, the expression of *TICRR* may be regulated by the m5C-driven methylation and then affects the tumor progression of LUAD.

Cell cycle and metabolism strongly interact in cancer. Oncometabolites can contribute to tumor growth by controlling gene expression and inhibiting homology-dependent DNA repair, which could increase DNA damage (36). Aerobic glycolysis could actively support function protein during DNA replication, especially in the G1 phase (39). This study suggests a positive correlation between cysteine- and glycolysis-related genes and *TICRR*. *TICRR* may regulate these metabolic pathways, supporting the energy demands of tumor growth and proliferation.

To confirm the stability of the genomic and DNA repair process, we subsequently analyzed the correlation between *TICRR* and DDR pathway genes. We found that all 34 DDR pathway-related genes show a positive correlation with *TICRR* expression, with the HR pathway gene *RAD54L* demonstrating the strongest association. Wang Y. et al. have found that high Rad54L expression promotes abnormal bladder tumor cell proliferation by changing the cell cycle and cell senescence (38). This study further demonstrates DDR pathway may affect the cell cycle by enhancing the *TICRR* expression, promoting LUAD tumor proliferation. In addition, we find that hsa-miR-27a-3p and hsa-miR-27b-3p are target miRNAs of *TICRR*, and the ceRNA network reveals some pairs of interacted genes, such as LINC01089/hsa-miR-27a-3p, hsa-miR-27a-3p/KCNQ1OT1/HSP90AA1, which help to regulate the *TICRR* expression. Guo, X. and M. Li have investigated that LINC01089 could affect the proliferation of GC cells by interacting with miR-27a-3p and upregulating the expression of *TET1* (40). Dong, Z. et al. demonstrate that upregulation of lncRNA *KCNQ1OT1* expression could regulate the cirRNA HSP90AA1 expression by sponging miR-27a-3p during NSCLC progression according to the cell lines validation experiment (41).

Zonderland et al. reported that depletion of *TICRR* resulted in the decoupling of DNA replication and cell-cycle progression from the early S-phase by silencing the *TICRR* gene in Hela cells (17). Silencing *TICRR* significantly affects several biological functions, including the cysteine metabolic process. Furthermore, it alters cancer-related pathways, such as suppressing the cell cycle pathway while enhancing the B/T cell receptor signaling pathway. These observations highlight the diverse role of the *TICRR* gene in regulating critical cellular processes and its potential implications in cancer-related pathways.

In pursuit of enhanced clinical applicability, we developed a *TICRR* risk model based on the *TICRR* network genes, accurately predicting LUAD patient survival, and its validity has been robustly confirmed in four independent GEO datasets. Moreover, we have integrated the *TICRR* risk model with relevant clinical characteristics, constructing a comprehensive nomogram that can effectively guide clinical practice. The findings of our study could provide valuable support for personalized treatment and patient management, enhancing prognostic assessments in LUAD patients.

We have confirmed that *TICRR* is overexpressed in LUAD, and we speculate that its impact on normal cell cycle processes may contribute to carcinogenesis and tumor progression. As a critical gene involved in the cell cycle, *TICRR* could activate the DDR pathway and allow the tumor cells to proliferate and avoid repairing DNA damage. *TICRR* is closely correlated with oncogenic metabolism and m6A/m5C regulation. Further experiment validations are required to uncover the functional mechanism of *TICRR* in LUAD. In summary, the high expression of *TICRR* causes more risk for LUAD patients and could become a potential prognosis biomarker and therapeutic target.

## 5 Conclusions

*TICRR* has been identified as a robust prognostic biomarker in lung adenocarcinoma (LUAD) due to its involvement in critical biological processes such as immune activation, cell cycle regulation, RNA modification, and tumor energy metabolism. The *TICRR* risk model and nomogram led a light on the clinical application value of *TICRR* gene. The comprehensive understanding of the functional relevance of *TICRR* in LUAD offers significant promise in facilitating its translation into a reliable therapeutic target and an effective prognostic indicator for LUAD.

## Data availability statement

The datasets presented in this study can be found in online repositories. The names of the repository/repositories and accession number(s) can be found below: Gene Expression Omnibus, https://www.ncbi.nlm.nih.gov/geo/, GSE72094, GSE50081, GSE13213, GSE30219, GSE41271, GSE42127, GSE126044, and GSE210129.

## Ethics statement

This study was approved by the Ethics Committee of the First Hospital of Putian City (No. 2023-044). The studies were conducted in accordance with the local legislation and institutional requirements. The participants provided their written informed consent to participate in this study.

## Author contributions

XZ: Conceptualization, Writing – original draft. LH: Formal Analysis, Writing – original draft. JG: Data curation, Formal Analysis, Writing – review & editing. CC: Formal Analysis, Writing – review & editing. YZ: Data curation, Writing – review & editing. JZ: Data curation, Visualization, Writing – review & editing. YRZ: Validation, Writing – review & editing. SL: Writing – review & editing. JS: Validation, Writing – review & editing. ML: Visualization, Writing – review & editing. HH: Conceptualization, Writing – review & editing.
