# Supplementary material for: TICRR serves as a prognostic biomarker in lung adenocarcinoma with implications in RNA epigenetic modification, DDR pathway, and RNA metabolism
Source: Front Oncol. 2023 Dec 13;13:1274439. doi: 10.3389/fonc.2023.1274439 (PMC10751343; doi:10.3389/fonc.2023.1274439)
Supplement: Supplementary file 1 [file DataSheet_1.docx]

Supplementary Material

## Supplementary Figures


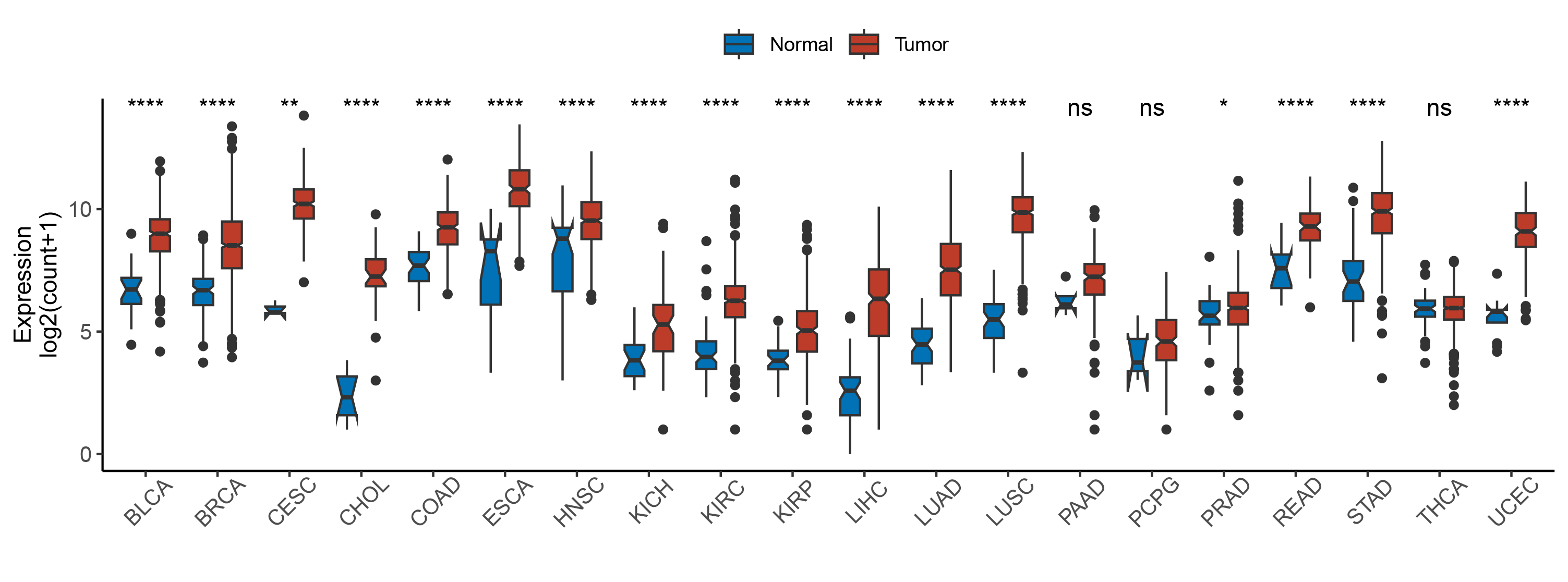


**Supplementary Figure 1. The different expression of *TICRR* between normal and tumor tissues in pan-cancer**

**
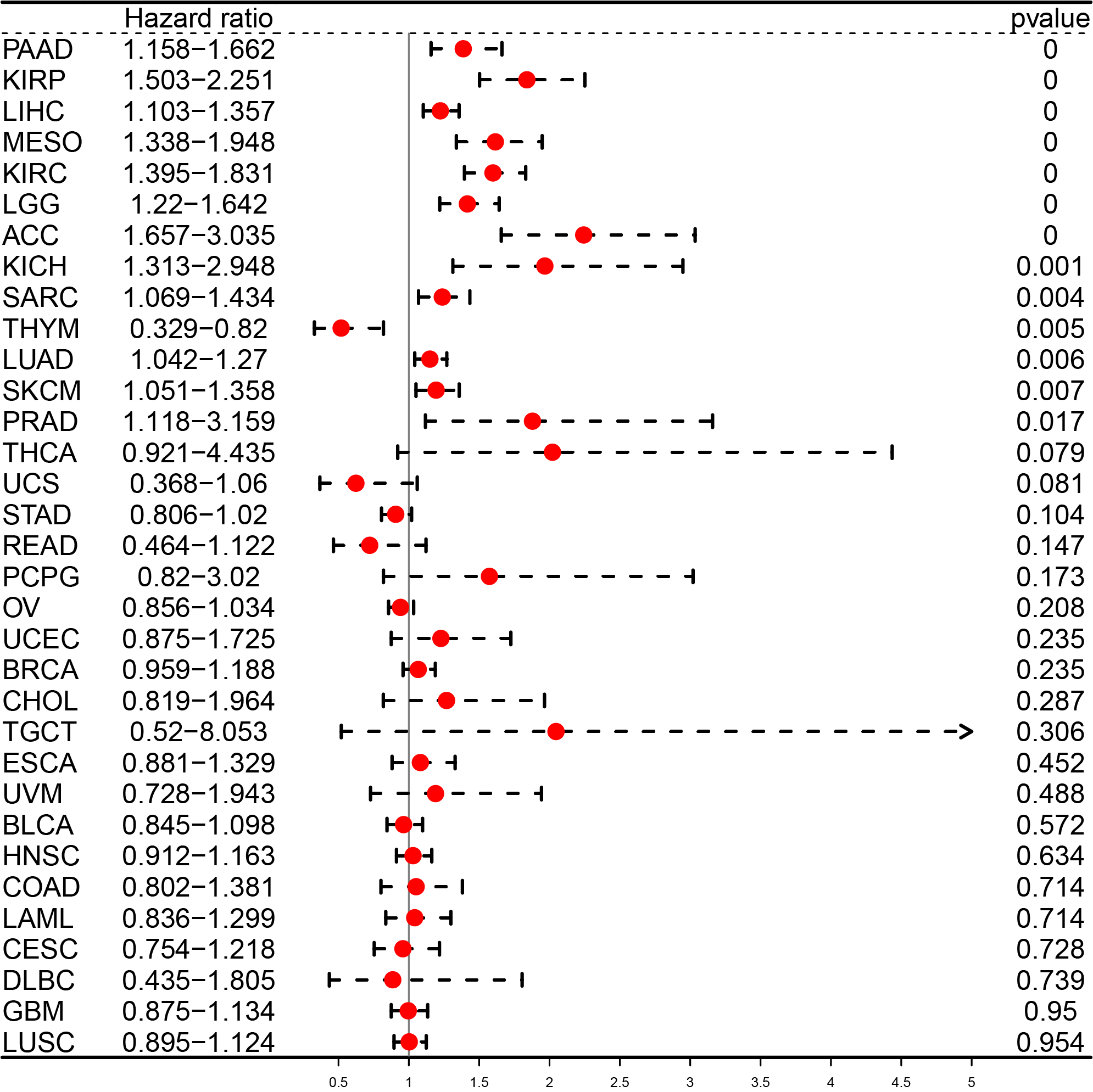
**

**Supplementary Figure 2.** **Cox proportional hazards regression analysis of *TICRR*.**


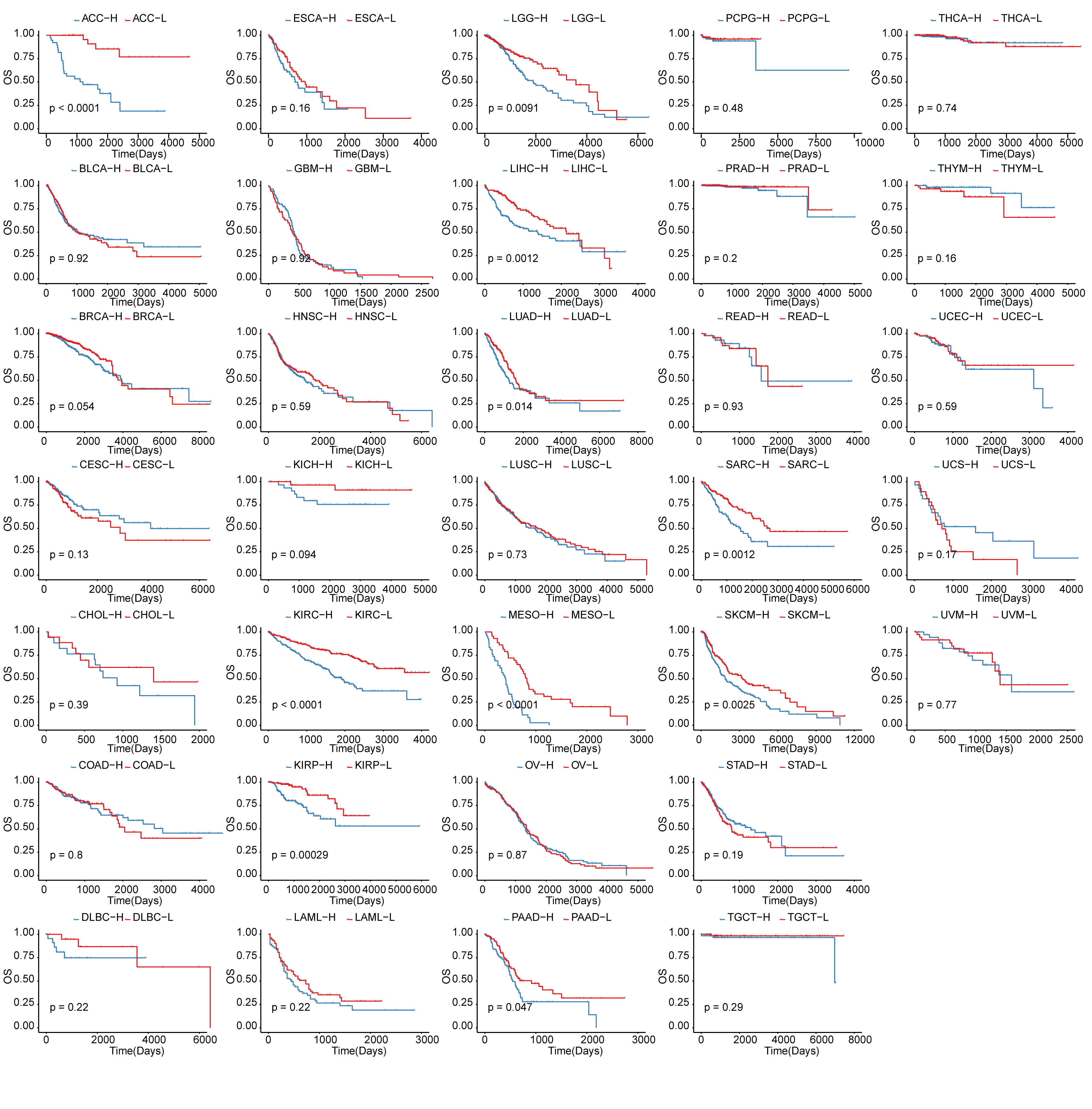


**Supplementary Figure 3.** **Survival analysis according to the expression of *TICRR*.** Red represents the low-risk group. Blue represents the high-risk group.


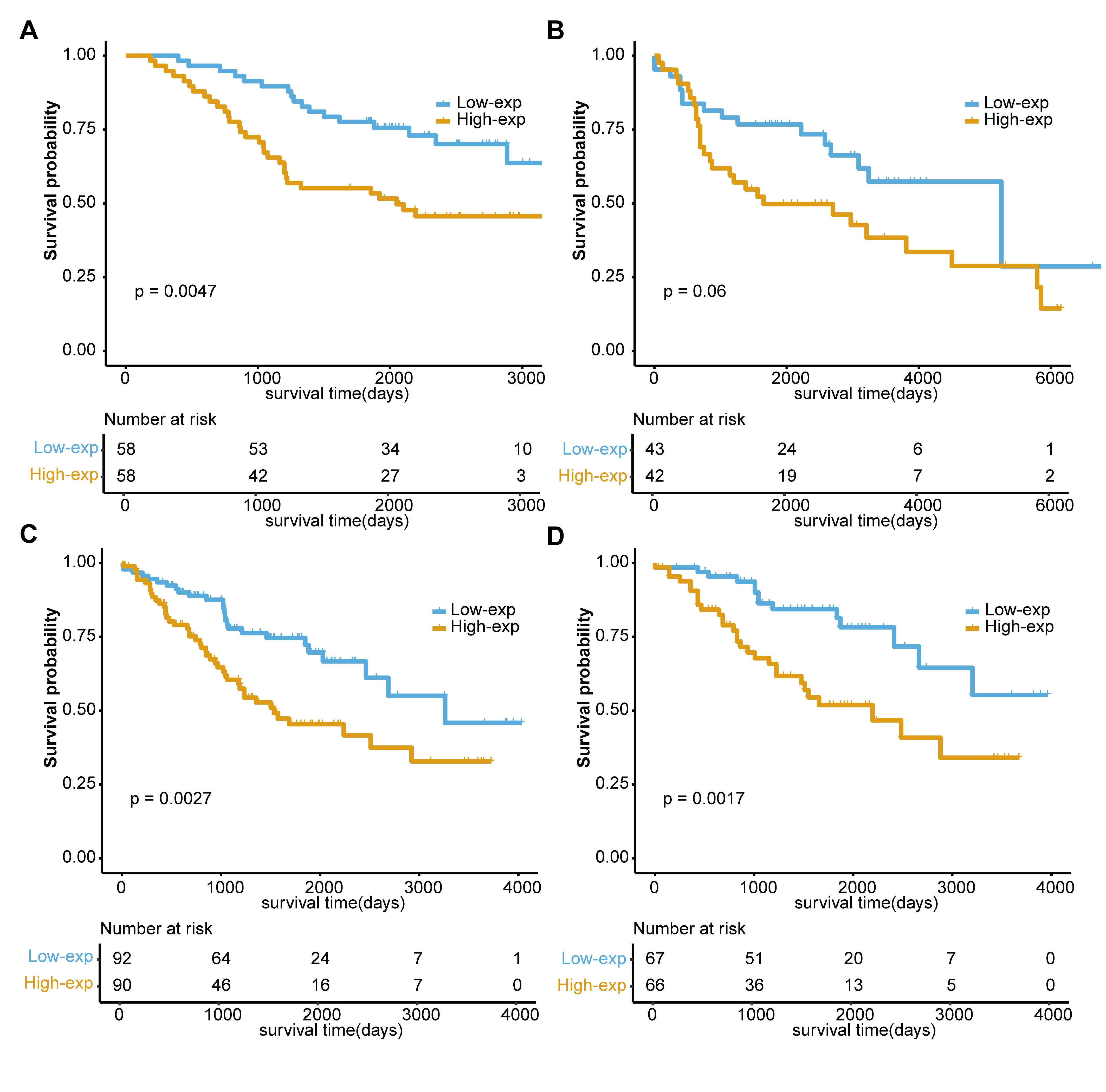


**Supplementary Figure 4.** **Prognosis value of *TICRR* expression in additional four GEO datasets.** Kaplan-Meier survival analysis for survival probability in (A) GSE13213, (B) GSE30219, (C) GSE41271, and (D) GSE42127 based on the TICRR expression.


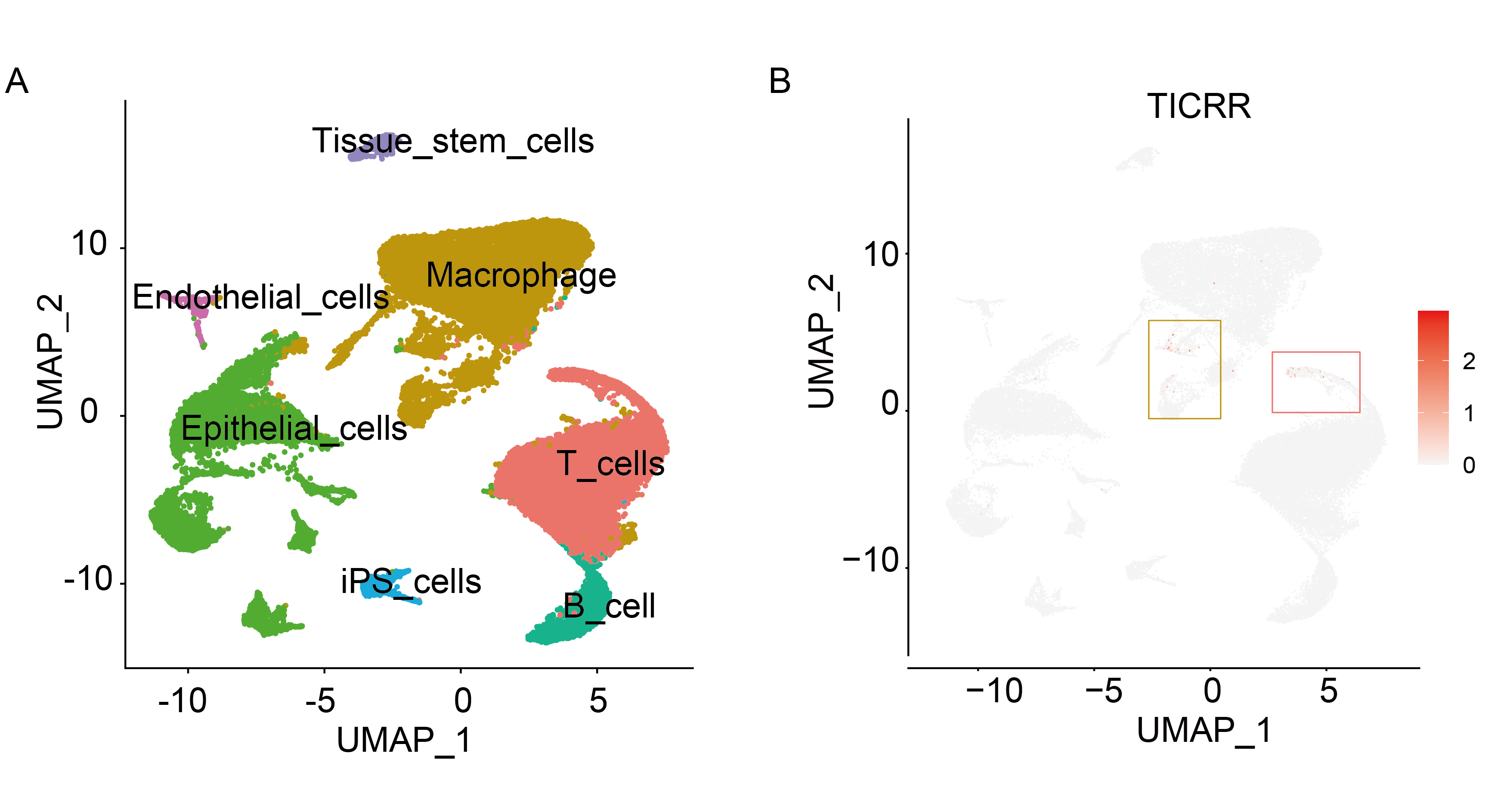


**Supplementary Figure 5. Single-cell analysis according to *TICRR* expression.** (A) The dimensionality reduction analysis performed by UMAP shows the distribution and dissimilarity of the seven cell types. (B) Distribution of TICRR in seven cell types.
